# White Matter Changes Along the Electrode Lead in Patients Treated With Deep Brain Stimulation

**DOI:** 10.3389/fneur.2018.00983

**Published:** 2018-11-21

**Authors:** Richard Erasmi, Oliver Granert, Dmitry Zorenkov, Daniela Falk, Fritz Wodarg, Günther Deuschl, Karsten Witt

**Affiliations:** ^1^Department of Neurology, Christian-Albrechts University of Kiel, Kiel, Germany; ^2^Department of Neurology, University of Cologne, Cologne, Germany; ^3^Department of Neurosurgery, Christian-Albrechts University of Kiel, Kiel, Germany; ^4^Department of Neuroradiology, Christian-Albrechts University of Kiel, Kiel, Germany; ^5^Department of Neurology and Research Center Neurosensory Science, Carl von Ossietzky University Oldenburg, Oldenburg, Germany

**Keywords:** Deep brain stimulation (DBS), electrode lead, white matter changes, MRI, Parkinson's disease (PD)

## Abstract

**Introduction:** Deep brain stimulation (DBS) is an established treatment for various movement disorders. There is little data available about the potential damage to brain parenchyma through DBS treatment. The objective of this study was to investigate the occurrence of signal changes on magnetic resonance imaging (MRI) in patients treated with DBS.

**Methods:** We retrospectively analyzed MRI scans of 30 DBS patients (21 patients with Parkinson's disease, 3 patients with dystonia and 6 patients with tremor) that had undergone additional MRI scans after DBS surgery (ranging from 2 months to 8 years). Axial T2 sequences were analyzed by two raters using a standardized lesion mapping procedure.

**Results:** 26 out of 30 analyzed patients showed hyperintense white matter changes surrounding the DBS lead (mean volume = 2.43 ml). Lesions were prominent along the upper half of the electrode lead within the subcortical white matter, with no abnormalities along the lower lead. Their volume was significantly correlated to the time from surgery to MRI and to the number of microelectrodes used in surgery, but was independent from underlying disease (Parkinson's disease, dystonia, tremor), target structure (STN, GPi, VIM), demographical data, or cardiovascular risk factors.

**Discussion:** White matter changes along the electrode leads in DBS patients are a frequent finding. These changes seem to evolve with certain latency after surgery and might be radiologically classified as a gliosis. Our findings identify the number of intraoperatively used microelectrodes as a risk factor in the formation of gliosis. Therefore, mechanical damage at the time of surgery and an individual tissue response might contribute to their evolution. Further studies are needed to define the exact mechanisms and their clinical impact.

## Introduction

Deep brain stimulation (DBS) is an effective and widely used treatment option for various movement disorders such as Parkinson's disease (PD), tremor, or dystonia(1, 2). Many efforts have been made to study the clinical side effects of chronic stimulation and the peri-procedural risks, also complications are well-characterized (3–7). However, there is limited data available on the potential damage to brain parenchyma through the implanted electrodes and leads in the course of DBS therapy. Histopathological autopsy studies on brains of DBS patients have been performed to assess the long-term structural effects of DBS electrodes and leads on brain tissue (8–13). The largest study, examining 26 post-mortem brains of DBS patients, showed only mild to moderate gliosis to DBS lead placement (13). Another study analyzed brain tissue of 10 patients treated with DBS up to 7.5 years and found minor axonal changes around the DBS electrode (14). Two retrospective studies analyzing postoperative MRI scans (with a maximum of 3 months after DBS surgery) have detected transient white matter changes, whose origin and significance remain uncertain (15, 16). Englot and colleagues found T2 signal hyperintensity surrounding DBS leads on postoperative MRI scans in 6.3% of 239 implants in 133 patients (15). The changes were considered to be non-infectious and non-hemorrhagic, but instead as a reactive and inflammatory tissue response. They were considered to be transient in nature and could not be correlated to a clinical manifestation of symptoms or worsening of stimulation effects (15, 16).

**Figure 1:**
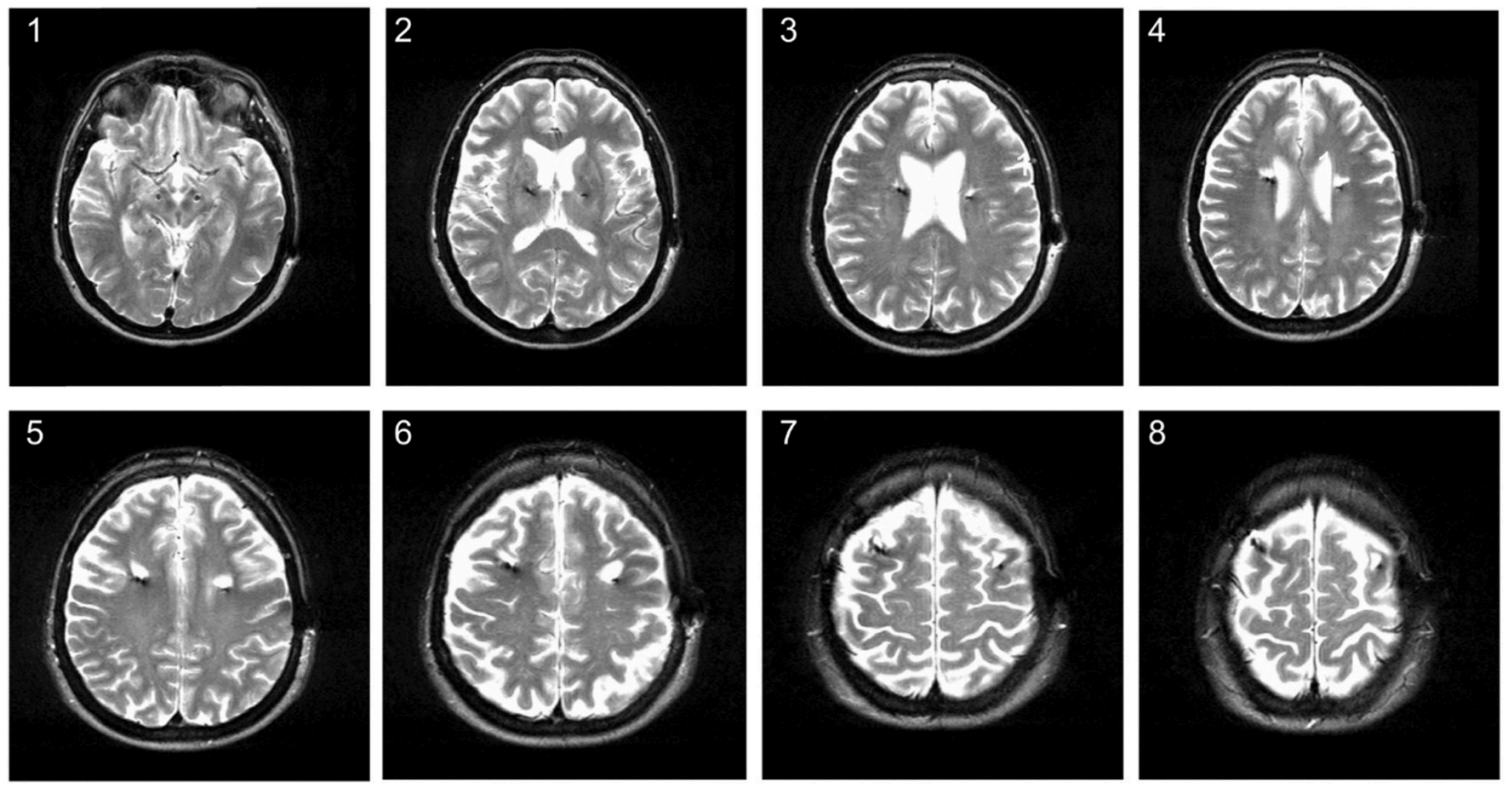
Hyperintense signal changes on MRI in a 58-year old patient treated with DBS for Parkinson's disease. The MRI was conducted about 3 years after STN-DBS surgery. The image shows hyperintense white matter lesions around the upper electrode lead on axial T2 series.

Over the past years, DBS patients in our center underwent MRI imaging during the follow-up for various reasons in addition to immediate postoperative imaging. MRI imaging has been demonstrated to be safe for patients with a (Medtronic®) DBS system, following certain precautions and restrictions (see 17 MRI GUIDELINES for Medtronic Deep Brain Stimulation Systems 2010). It can provide excellent anatomical resolution and is routinely being used for postoperative imaging in order to detect complications and to confirm electrode placement (18, 19). Noticeably, we have observed white matter changes around the electrode lead (for an example see Figure 1). This prompted us to assess the occurrence of MRI changes in patients treated with DBS in a retrospective study to obtain more information about potential effects of DBS on brain parenchyma.

## Methods

This study was approved by the local ethics committee. We searched our clinical database to identify all DBS patients (with PD, dystonia, or tremor) that had undergone one or more MRI imaging in addition to the routine postoperative imaging at least 60 days after surgery. Included were patients who had received DBS electrode implantation between 2001 and 2015 with a Medtronic system (Soletra®, Kinetra®, or Activa®) according to published surgical procedures (20, 21). Patients had consented in written form that their clinical data is to be used for research purposes. In total, we identified 86 patients with additional MRI imaging out of a total number of 678 DBS patients. The following exclusion criteria were defined: (i) postoperative complications (e.g., bleeding, infarction, or oedema), (ii) surgery for explantation or revision, (iii) severe leukoencephalopathy (as DBS-induced changes cannot be identified).

As the main outcome parameter we defined new white matter lesions along the DBS lead on T2 weighted imaging. We chose the T2 sequence as it was part of a standardized MRI protocol, was present in all of our patients, lesions could be clearly delimited from the surrounding tissue and the hypointense electrode artifact. For the available routine imaging studies, the field of interest was often limited to the region around the target point for safety reasons. Thus, patients had to be excluded if the region around the cortical entry point was not visible during the T2 weighted image sequence.

For our main analysis, we conducted a lesion-mapping procedure using MRIcron software (Version 6.6 2013 by Chris Rordon, McCausland Center for Brain Imaging, Columbia SC, USA) on T2 weighted sequences, produced by a 1.5 Tesla MRI scanner with slice thickness ranging from 2.2 to 5.2 mm. Two independent raters, blinded to the clinical data of the patients, were instructed to map lesions in a semi-standardized way (for the criteria see Table 1 and for an example see Supplementary Figure 1). Each rater was allowed to choose a contrast that enabled them to discriminate lesions from intact tissue. Each rater scored the quality of the MRI scan according to the criteria “good quality” (defined as scans free of artifact and the lesion criteria could be applied without restrictions), “moderate quality” (defined by the presence of some artifacts that lowers overall interpretation but the defined lesion criteria could still be applied), and “poor quality” (defined by the presence of artifacts or vascular lesions surrounding the electrode preventing the application of lesion criteria). Both raters showed an accordance of 100% for scans classified as “poor quality.”

**Table 1:** Lesion criteria.

**A lesion (considered eligible for mapping) was defined as a T2 hyperintense signal change that meets following criteria:**
•has direct contact to the hypointense DBS lead/ electrode artifact. •can be clearly distinguished from surrounding structures and the hypointense DBS lead/ electrode artifact. •exceeds in its (separate) measurement the diameter of the DBS electrode/ lead artifact. •cannot be explained (better) by another underlying or pre-existing pathology (e.g., microvascular lesion, infarction or any other findings on pre- and postoperative imaging).

The total lesion volume was then calculated in ml for each patient and each side. Further image data preprocessing (normalization) and analysis were done with the SPM8 software (http://www.fil.ion.ucl.ac.uk/spm/) on Matlab 7.7.0 (MathWorks) and with some additional in-house software components developed to determine electrode lead position and to locate the extent of lesion in proportion to the lead. To locate the lead trajectory, we manually placed between 4 and 5 control points along the midline of the electrode artifact (separately for the left and right electrode) within the T2 images. We then utilized a 3D least squares optimization procedure to determine the center of the electrode in the individual patient and extracted the intensity profile along this lead trajectory. The area around the electrode contacts is characterized by a steep intensity dip in T2 signal, which we used to manually position the four contacts according to the known contact distances from the data sheet of the implanted electrodes (22). In order to measure the area of gliosis along the lead trajectory, the mask images were resliced separately for each side in planes orthogonal to the lead in 2 mm steps providing slices with a fixed distance to the lead contacts.

We additionally used the SPM normalization parameters to transform the gliosis masks into standard MNI space. The normalized volumes from all patients were then combined by calculating voxel-wise mean values. This mean volume image was finally visualized by mapping the color coded mean values in a range from 0.2 to 1 (corresponding to 20–100% local gliosis occurrence) onto the standard single subject T1 template to show the localization of the gliosis in standard MNI space.

Final statistics were calculated using R (version 2.15.2, Copyright © 2012 The R Foundation for Statistical Computing). A paired *t*-test was used as the standard non-parametric and Pearson correlation as the standard parametric method; if deviation from a normal distribution was observed, the Spearman correlation was used. Direct postoperative imaging was screened for complications such as bleeding and infarction and for changes around the DBS electrode lead.

## Results

Out of the 86 eligible DBS patients, 56 had to be excluded (5 due to postoperative complications, 13 due to second surgery, 2 because of severe leukoencephalopathy, and 36 because of insufficient T2 imaging with slices at electrode entry lacking at the >60 day postoperative imaging). Of the 30 remaining patients, 21 had PD, 3 had dystonia and 6 had tremor (5 with essential tremor, 1 with orthostatic tremor). All patients were implanted bilaterally with a Medtronic System (Kinetra® oder Activa PC®) in our center into the subthalamic nucleus (STN, *n* = 20), the globus pallidus pars internus (GPi, *n* = 2) or the nucleus ventralis intermedius of the thalamus (VIM, *n* = 8). Mean age at surgery was 59.9 years and the time interval between surgery and MRI imaging ranged from 68 days to 98.2 months (≈ 8.2 years) with an average of 37.8 months (≈ 3.1 years). The most common reasons for MRI imaging were missing or weaning effect of stimulation (*n* = 7), hardware infection (*n* = 3), suspected stroke (*n* = 5), deterioration of gait and cognition (*n* = 4), a cerebellar syndrome in tremor patients (*n* = 5), and other reasons (*n* = 6).

**Figure 2:**
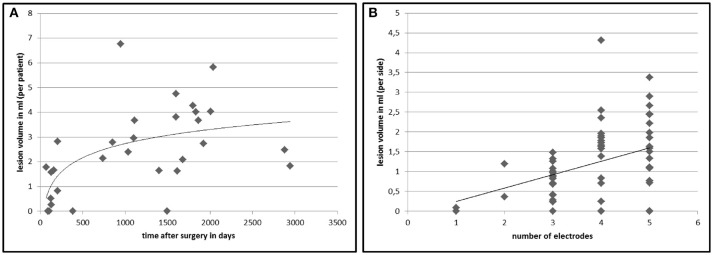
Lesion volumes in correlation to time after DBS surgery and number of electrodes used in surgery. The chart displays the calculated total lesion volumes of each patient on the y-axis plotted against the time between surgery and MRI imaging **(A)**. **(B)** shows the lesion volume surrounding an electrode (*n* = 60) in relation to the number of inserted microelectrodes at surgery to find the best place for the final electrode. The lines represent a non-linear (left, *r* = 0.58, *p* < 0.005) and a linear (right, *r* = 0.38, *p* < 0.05) regression curve.

In 26 out of 30 patients (86.7%) we found white matter changes around the DBS lead that fulfilled the lesion criteria (for criteria see Table 1). Inter-rater variability for the lesion mapping was *r* = 0.92 (correlation coefficient, Pearson correlation). The mean volume of the lesions per patient was 2.43 ml (range: 0.26–6.76 ml). Lesion volumes for right and left trajectories were not different with a mean volume of 1.24 ml for all left and 1.19 ml for all right trajectories (*p* = 0.72). We analyzed MRI scans with different slice thicknesses ranging between 2.0 and 5.2 mm. We found no significant correlation between slice thickness and total lesion volume. This analysis did not exclude a bias derived from an analysis of various slice thickness. However, this analysis shows that data from various slice thickness does not systematically influence the lesion volume. There was no significant difference in lesion volume between patients with PD and tremor (*p* = 0.27, statistical calculation is likely to be underpowered due to the small number of tremor patients with *n* = 6) or a correlation to the target structure (STN vs. VIM, *p* = 0.68). Furthermore, there were no correlations of the lesion volume with demographical data, such as age at the time of surgery (*p* = 0.78), gender (*p* = 0.16), disease duration (*p* = 0.88), or the existence of cardiovascular risk factors like diabetes or hypertension (*p* = 0.75). However, lesion volume was positively correlated to the time from surgery to MRI imaging (*p* < 0.005, *r* = 0.58) and to the number of electrodes used in stereotactic surgery for neurophysiological assessment (*p* < 0.05, *r* = 0.38; see Figure 2).

**Figure 3:**
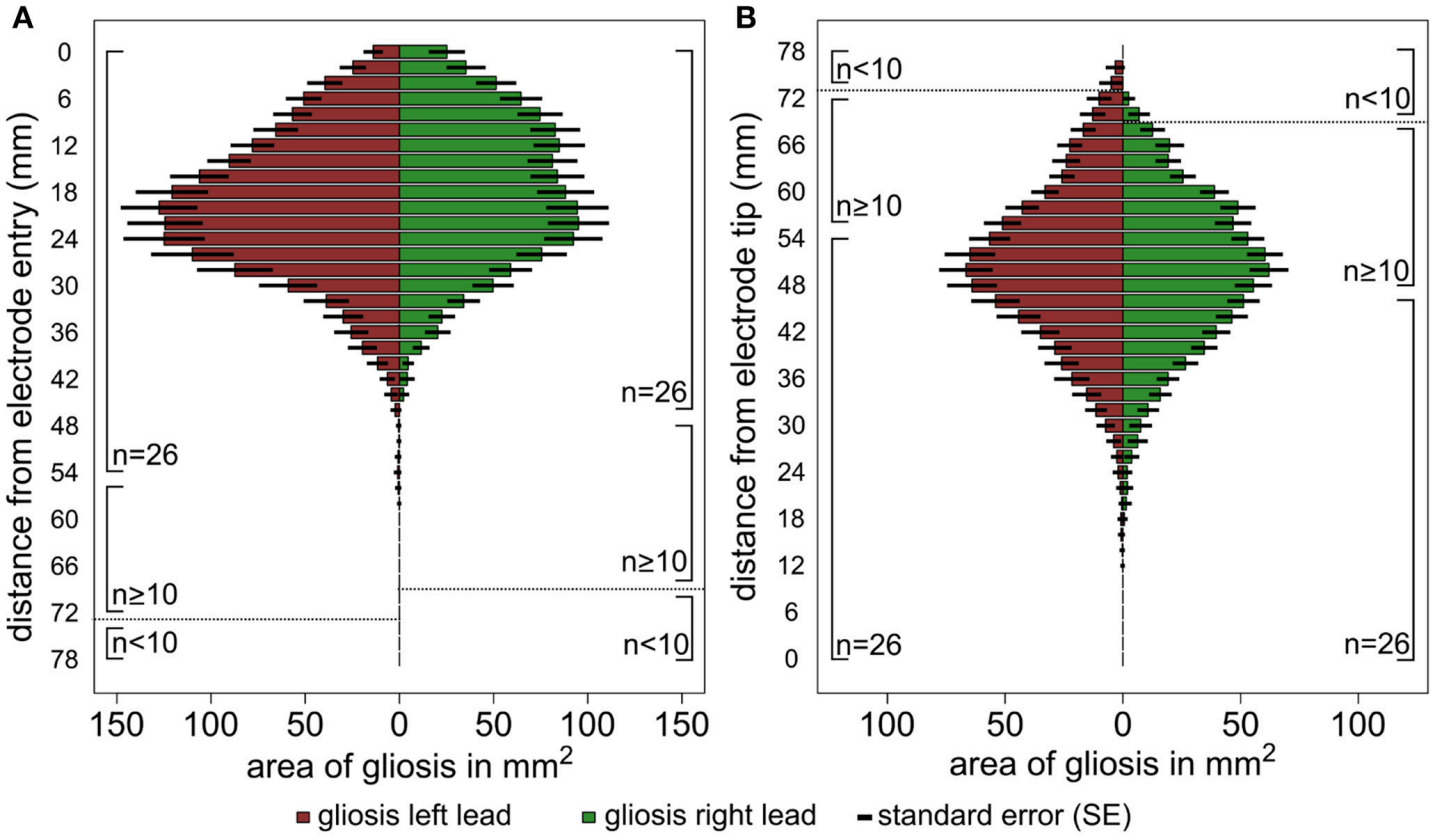
Graphic illustration of the results of the lesion mapping procedure. The horizontal columns represent the average area of the gliosis in mm2 measured at an orthogonal angle with the DBS trajectory. The results are shown for all left trajectories (red) and right trajectories (green) separately, the number of available measurements (n) is shown at the side of the columns. **(A)** Shows the area measurements of the gliosis with regard to the distance from the cortical entry point of the lead and **(B)**. with regard to the distance from the electrode tip. The interrupted line indicates a relevant reduction of the sample size (*n* < 10).

The results of the lesion mapping procedure of the 30 patients are displayed in Figure 3. Figure 3A shows the averaged area of the lesions at an orthogonal angle with the DBS trajectory with regard to the distance from the cortical entry point of the lead (i.e., entry point is mapped to 0 mm). Due to the different lengths of the electrode within the brain, the lesion mapping loses accuracy toward the electrode tip. The lesions begin at the entry point of the electrode lead with its maximum extent between ~12 and 30 mm below the entry point and a clear tendency to decrease toward the lower lead. Figure 3B shows the same measurements referenced to the electrode tip (i.e., electrode tip is mapped to 0 mm), thus losing accuracy toward the entry point. No relevant lesion was observed around the electrode tip and the lower lead, whereas at about 35 mm above the electrode tip the extent of the lesion grows toward a maximum of around 40 to 60 mm above the electrode tip, decreasing in size again toward the cortical entry point. Supplemental Figures 4A–C show the lesion volume according to different target areas (STN, VIM, GPi).

**Figure 4:**
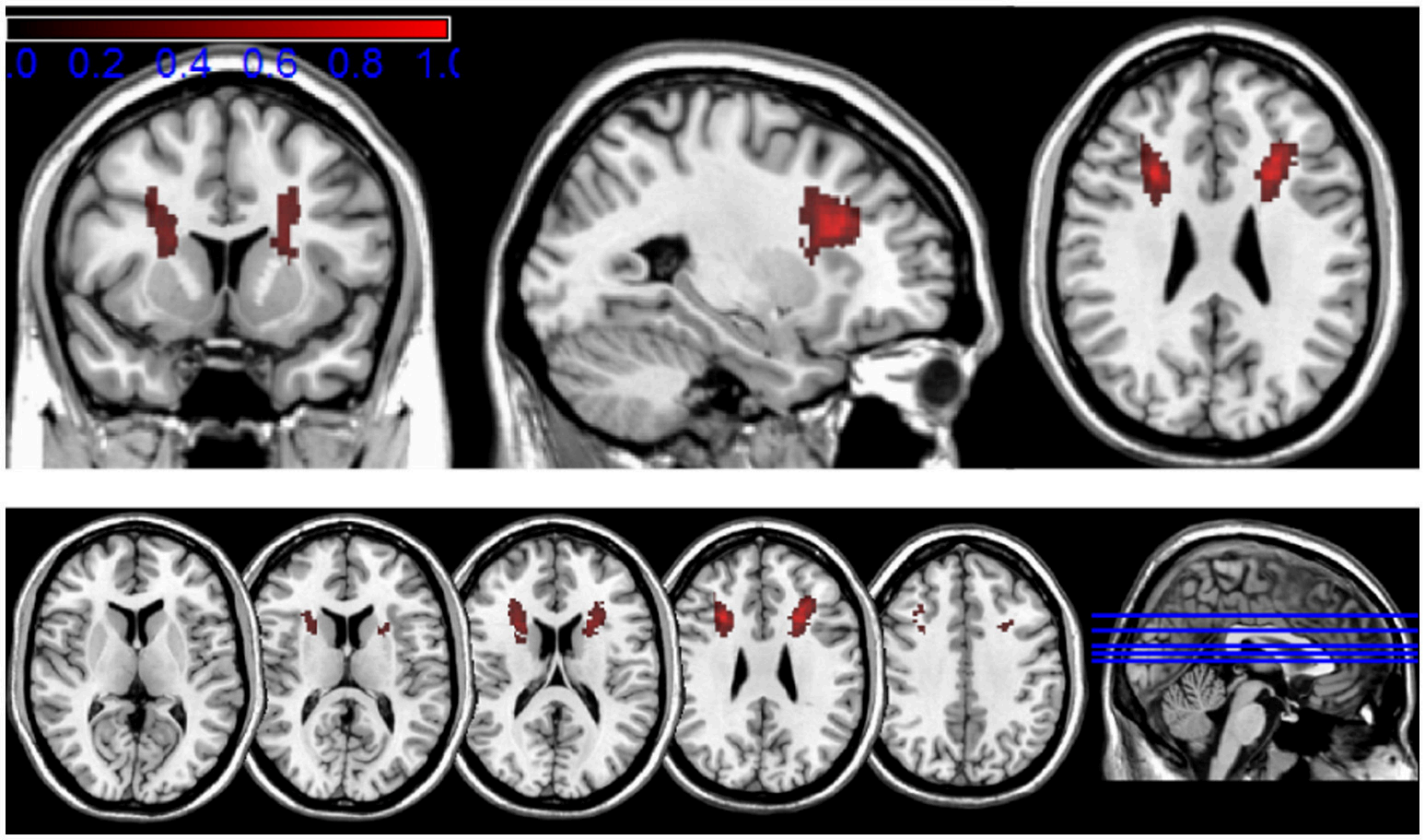
Transformation of the mapped lesions in standard MNI space. The results of the lesion mapping are color-coded in a range from 0.2 to 1 (corresponding to 20–100% local gliosis occurrence) to roughly visualize the anatomical distribution of the gliosis in the human brain.

Figure 4 shows the localization of the lesions in standard MNI space, color-coded to roughly visualize the anatomical distribution of the gliosis on the human brain. The main extent of the gliosis is located at the level of subcortical, frontal white matter structures, affecting mainly the anterior and superior corona radiata (using the JHU white matter atlas regions as orientation).

Both approaches identify the main extent of white matter lesions along the upper half of the DBS lead involving mainly frontal subcortical white matter structures and reaching its maximum expansion between 12 and 35 mm below the entry point. Both approaches show a decrease of lesion expansion in direct proximity to the entry point. No relevant lesion is detected on the level of subcortical gray matter structures around the lower lead and around the electrode tip in the target region.

A small group of patients (*n* = 6) had undergone more than one additional MRI. In half of the patients we found an increase of lesion volume from the previous to the later MRI, in two cases lesion volume was stable, and in one patient a decrease of lesion volume was measured (see Supplemental Figure 3). Patients' characteristics are shown in Table 2 and data is shown in the Supplemental Figure 3.

**Table 2:** Patient characteristics.

**N**	**Sex**	**Diagnosis**	**Age at surgery (yrs)**	**Duration of disease at surgery (yrs)**	**Target**	**Time from surgery to MRI (m) (1) (2)^a^**	**Number of microelectrodes r/l**	**Position of the active Electrode left side^b^(y/x/z)**	**Position of the active electrode right side^*b*^(y/x/z)**
1	M	Dystonic tremor	31	n.a.	VIM	4	3/3	na	na
2	F	Dystonia	52	11	GPI	7	3/3	21.6/−3.7/−5,5	−19.8/−3.3/−7.3
3	F	Dystonia	69	9	GPI	13	3/4	21.6/−4.7/−5.5	−19,8/−4.3/−6.3
4	F	PD	55	7	STN	3	5/5	na	na
5	F	PD	48	8	STN	53; 60	5/5	10.2/−3.7/−6.3	−11.8/−4.3/−3.6
6	M	PD	74	3	STN	37	5/na	12.1/−3.8/−3.6	−13.7/−2.8/−5
7	M	PD	52	n.a.	STN	68	5/5	12.2/−1.2/−4.5	−12.2/−1.6/−2.1
8	F	PD	54	1	STN	98	5/5	11.3/−2.6/−2.2	−12.4/−2.2/−2.1
9	F	PD	75	8	STN	5; 23	1/4	13/2,5/−2.7	−12.1/−1.3/−2.4
10	M	PD	58	12	STN	56	2/3	12.5/−2.3/−3.6	−12.3/−3.6/−3.1
11	F	PD	63	7	STN	28	4/4	13.4/−0.8/−2.7	−11.7/−1.8/−2.6
12	M	PD	73	4	VIM	35	4/5	na	na
13	F	PD	59	5	STN	67	4/3	12.6/−1.4/−5.1	−10.9/−1.6/−3.3
14	F	PD	55	12	STN	31; 36	5/4	13.3/−0.7/−3.3	−9.6/−1.6/−4.2
15	M	PD	43	6	STN	54	372	11.3/−1.1/−4.8	−12.2/−1.6/−2.1
16	M	PD	68	21	STN	3; 9	5/5	11.6/−0.9/−4.2	−13.4/0.3/−4.2
17	M	PD	54	7	STN	37	5/4	11.1/−1.2/−2.7	−10.6/−1.6/−4.9
18	M	PD	65	6	STN	4	4/5	11.7/0.5/−4	−12.8/0.3/−4.1
19	M	PD	71	n.a.	STN	4	3/4	11.4/1.2/−3.6	−12.2/−0.6/−3.4
20	M	PD	59	26	STN	47	3/3	13.2/0.4/−2.8	−11.6/−0.8/−3.1
21	F	PD	56	n.a.	STN	60	5/5	11.9/−0.9/−3.3	−10.5/−2.4/−6.3
22	F	PD	66	10	STN	64	4/3	12.5/−0.8/−3	−11.5/1.6/−3.6
23	F	PD	50	14	STN	96	4/5	10.8/−3.5/−5.5	−11.9/−0.4/−3.3
24	M	PD	69	10	STN	50	1/4	11.4/−2/−5.4	−9.2/−3.4/−5
25	F	Tremor	71	10	VIM	53	5/5	13.4/−6.8/2.4	−14.6/−6.8/2.5
26	F	Tremor	40	19	VIM	62	4/4	na	na
27	F	Tremor	77	2	VIM	7	5/5	11.5/−5/1.0	−12.1/−8.5/1.4
28	F	Tremor	69	14	VIM	25; 63	4/4	11.3/−4.6/2.3	−11.2/−6.1/1.5
29	F	Tremor	53	2	VIM	2; 16	3/3	12.5/−6/2.1	−13.3/−7/1.1
30	F	Tremor	68	n.a.	VIM	25	3/3	13/−5.9/3	−11.6/−7.9/2.4

a *Time form surgery to MRI in months, in 6 patients (5, 9, 14, 28, and 29) two MRI scans were performed*.

b *Position of the active electrode is given according individual midpoint of the ac-pc line*.

## Discussion

Deep brain stimulation is an effective and widely used treatment for PD, dystonia, and tremor. Despite powerful studies about efficacy, long-term outcomes, and side effects, limited data exists on the possible effects of DBS on brain parenchyma. We systematically assessed the occurrence of MRI changes in patients treated with DBS and found hyperintense T2 lesions along the DBS leads in 26 out of 30 patients (≈ 87%), which can be radiologically classified as gliosis. Lesions were most pronounced along the upper half of the DBS lead involving subcortical white matter structures. Their volume was found to correlate significantly with time after DBS surgery and with the number of electrodes used for neurophysiological assessment in surgery, while the development of lesions does not seem to depend on the type of underlying disease, target structure, or demographical data such as gender, age at time of surgery, disease duration, or existence of cardiovascular risk factors.

Our findings indicate that the initial damage to the brain during surgery might play a major role in the formation of the lesions. This thesis is mainly based on a positive correlation between the number of electrodes used (for neurophysiological assessment and clinical testing) in surgery and the volume of lesions. This positive correlation leads us to assume, that the amount of initial damage to the brain through the penetrating guide tubes and microelectrodes is one of the contributing factors for the formation of white matter lesions in the course of DBS treatment. Notably, the pattern of the observed lesions can be considered as a support for this thesis. As previously stated, lesions were found to evolve along the upper half of the electrode lead, involving mainly subcortical white matter structures up to about 4 cm below the entry point with virtually no lesions below that level. For DBS surgery in our, as well as, inmany other centers, multiple combined micro- and macroelectrodes (usually 3–5 in number) are used for neurophysiological assessment of the target region and for evaluation of stimulation effect. To guarantee their safe passage through the brain parenchyma on the planned path, they are guided by rigid guide tubes which penetrate into the brain to a level of about 1–2 cm above the target point. Considering these circumstances, one possible explanation for the distinct pattern of lesions might be the presumably larger amount of damaged brain parenchyma through the guiding tubes (diameter ≈1.7 mm) in comparison to the significantly thinner electrodes (diameter ≈50 μm), which penetrate only the last 1–2 centimeters into the target region. The apparent decrease of lesion size in immediate proximity to the entry point might be explained by a mere lack of potential “damageable” brain parenchyma as the entry point is usually located on top of a gyrus structure and by inaccuracy of MRI data in that region. The distinct pattern of gliosis might also be explained partially by a higher vulnerability of white matter in comparison to gray matter. Altogether, the amount of initial damage to brain parenchyma through the penetrating electrodes/guide tubes can be proposed as a major contributing factor for the later evolution of gliosis.

The initial surgical damage does not seem to be the only contributable factor for the evolution of gliosis. Our data indicates the delayed evolution of new lesions in the course of DBS treatment, occurring most likely within the first 12–24 months after surgery and possibly reaching a more or less stable size at some point. Neither comparable pre-existing lesions nor immediate postoperative complications were detected on pre- and 1-day standard postoperative MRI. Also a positive (most likely non-linear) correlation was found between volume of the lesions and time after DBS surgery. Interestingly, there were a couple of patients with considerable white matter changes after only a few months of DBS treatment. On the other hand there were also patients, who even after several years of DBS only had very small lesions or none at all. This variable evolution of gliosis leads us to suspect additional contributing factors. Possibly, the evolution of gliosis depends on a number of factors, such as surgical variables (e.g., the coagulation of bridging veins—which is only performed when necessary in DBS surgery), an individual tissue vulnerability, and response to foreign materials and to mechanical forces, such as shearing/ shifting forces caused by movements of the brain against the fixated DBS lead at the skull. (Note: In all our patients an Ethiloop-covered mini plate was used for the fixation of the lead at the skull).

On the other hand, the distinct pattern of lesion—sparing of the lower lead and the target region—indicate that neither material nor the chronic electrical stimulation are responsible for their formation.

There is a discrepancy between our results and the lack of corresponding findings on histopathological post-mortem tissue. Only one of the post-mortem studies (14) found tissue changes at the entry zone of the electrode in a few patients while we found frequent changes on MRI in our study population. It is unlikely that the lesions are an isolated phenomenon of our center since we share the same standard as in many other DBS-centers. A mere artifact of MRI-imaging is highly unlikely because (1) The lesions can not only be identified on T2 but also on Flair series, (2) The lesions can be clearly discriminated from the hypointense electrode artifact and (3) Their variable shape. Nevertheless, it remains open to debate whether the observed MRI changes reflect an irreversible structural damage of brain parenchyma. This should be evaluated in studies focusing on an histopathological analysis. There are some limitations of the present study. Firstly, this was a retrospective study. MRI imaging quality was limited due to restrictions for DBS patients in the MRI scanner and movement artifacts caused by the underlying disease. Our analysis was based on axial T2 weighted sequences with variable image quality, variable slice thickness and partly inaccurate identification of the entry point of the lead. Thus, the precision of the lesion mapping was limited, especially in the cortical region around the entry point of the DBS lead. We tried to minimize potential inaccuracy in the mapping of the lesions by using two independent raters and by including only T2 sequences with moderate to good image quality and complete visibility of the DBS electrode lead including the cortical entry region. The high drop-out rate was driven by incomplete MRI data sets. Given the fact that the clinical problem that triggered MR imaging was mostly related to the location of the stimulation electrodes, the MRI scans were stopped at the level of the centrum semiovale in 36 cases. To fully assess the lesion load surrounding the leads, we had to include only scans that covered the whole brain.

Secondly, a bias may be induced as only patients with a relevant postoperative problem were scanned. This involved patients with low response to stimulation, weaning of stimulation effect, or other neurological problems (such as symptoms of stroke, gait disturbances, or cognitive decline).

Given the retrospective nature of the study design and the lack of sufficient clinical data at the time point of the MRI scans, we were unable to report the clinical significance of the white matter lesions surrounding the electrode trajectories. The majority of the lesion load evolves within the first 3 years (see Figure 2B). A slight but significant decline in verbal fluency performance and Stroop Test performance was reported after STN-DBS (7, 23, 24), most often evaluated after 6 months after surgery. Interestingly a study with a shifted starting design demonstrated deficits in category fluency performance in the stimulation OFF group (25) 3 months after surgery. These findings point to the fact that a micro-lesion at the level of the target point, the trajectory of the electrode lead or the evolution of a lesion surrounding the electrode causes such a deficit in close relation to the time of surgery (26). Several fiber-tracts such as the uncinated fasciculus and the fronto-striatal tract pass the lesioned subcortical white matter region surrounding the electrode leads and are part of a network responsible for category fluency performance (27, 28). Three arguments speak against an association between subcortical lesions described here and neuropsychological disturbances. Firstly, electrode leads for VIM and GPi DBS are very close to the location of lead trajectories targeting the STN. However, specific neuropsychological deficits are only described in PD, not in ET or dystonia. This finding indicates a disease (Parkinson's disease) or target (STN) specific role in cognitive changes after surgery (29). Secondly, previous studies investigating the impact of lead trajectories and cognitive decline after DBS found a significant association between a lesion of the caudate nucleus and verbal working memory decline (22), whereas no specific white matter trajectory responsible for cognitive changes was described. Thirdly, cognitive changes after STN-DBS were described after 3–6 months (7, 23, 25), whereas the evolution of the lesions surrounding the electrode lead shows its maximum about 3 years after surgery (see Figure 2A). Considering the fact that Aybeck al. (30) found no increased risk for dementia in STN-DBS treated PD patients in the first 3 years after surgery, the described changes may have a limited effect on global cognition. In conclusion, we report on possible brain parenchyma changes in patients with DBS. We demonstrate frequent hyperintense T2 MRI changes along the upper part of the DBS lead, which seem to evolve with certain latency after surgery and can be radiologically classified as gliosis. Our results indicate a primary surgery-related mechanism in the formation of the lesions, suggesting the number of penetrating guide tubes/microelectrodes used in surgery as a major causal factor. This finding might support a cautios and step-wise approach toward the number of microelectrodes used in DBS surgery. The delayed evolution of gliosis and their variable size point to the existence of other contributing factors, which cannot be identified in this study. The exact causes and the clinical significance of those findings remain uncertain and further studies are needed to reveal possible neuropsychological signs and symptoms associated with subcortical white matter lesions surrounding the electrode lead in patients with DBS.

## Disclosure

RE received training support from Medtronic and Merz, travel grants from MDS (Movement Disorder Society), and lecture fees from Medtronic. OG reports no financial disclosures. DZ received speaker fees from Medtronic and training support from Allergan and Medtronic. DF received lecture fees from Medtronic. FW received speakers honoraria from Penumbra Europe GmbH. GD has received lecture fees from Medtronic and Desitin and has been serving as a consultant for Medtronic, Sapiens, and Boston scientific. He received royalties from Thieme publishers. He is a government employee and receives through his institution funding for his research from the German Research Council, the German Ministery of Education and Health and Medtronic. KW reports grants from German Research Council and the German Ministery of Education and Health.

## Author contributions

RE collected and reviewed the data, performed the lesion mapping, and wrote the first draft of the manuscript. OG was mainly responsible for the developments and the application of the used methods and executed the statistical analysis. DZ was the second rater in the lesion mapping procedure. DF and FW critically reviewed the article and contributed to the manuscript equally. GD and KW designed the conception of the study and the manuscript, and participated in writing the first draft.

### Conflict of interest statement

The authors declare that the research was conducted in the absence of any commercial or financial relationships that could be construed as a potential conflict of interest.
