# Supplementary material for: White Matter Changes Along the Electrode Lead in Patients Treated With Deep Brain Stimulation
Source: Front Neurol. 2018 Nov 21;9:983. doi: 10.3389/fneur.2018.00983 (PMC6259286; doi:10.3389/fneur.2018.00983)
Supplement: Supplementary file 1 [file Data_Sheet_1.pdf]

Supplementary material for

“White matter changes along the electrode lead in patients treated with deep brain stimulation”, Front. Neurol. - Movement Disorders

R. Erasmi[1,2], MD, O. Granert[1], D. Zorenkov[1], MD, D. Falk[3], MD, F. Wodarg[4], MD, G. Deuschl[1], MD, PhD, K. Witt[1,5] MD

[1] Department of Neurology, Kiel University, Germany

[2] Department of Neurology, University of Cologne, Germany

[3] Department of Neurosurgery, Kiel University, Germany

[4] Department of Neuroradiology, Kiel University, Germany

[5] Department of Neurology and Research Center Neurosensory Science, Carl von Ossietzky University Oldenburg, Germany

Supplementary material for **DOI:** 10.3389/fneur.2018.00983

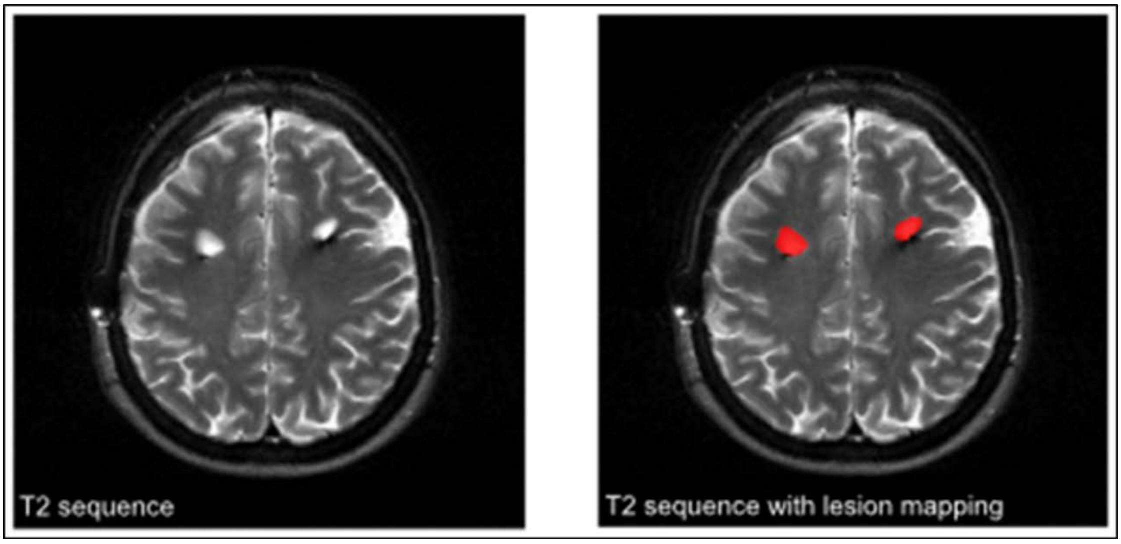

Supplemental Figure 1 | Example of the lesion mapping. A hyperintense T2 signal change around the electrode lead in a patient with DBS (left image) is mapped using MRIcron and marked in red (right image).

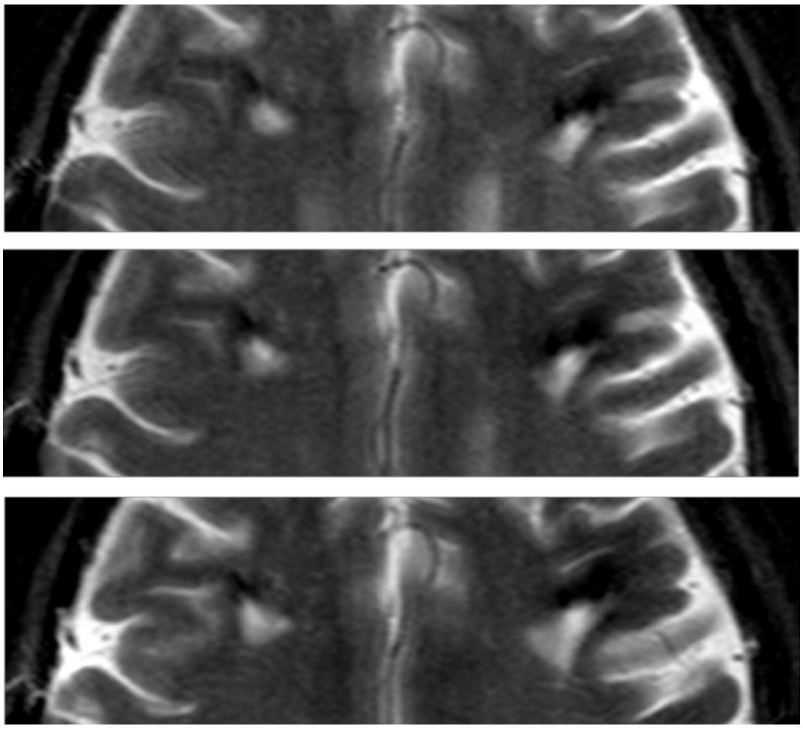

Supplemental Figure 2 | Example of an increasing white matter lesion in a PD patient treated with STN- DBS. MRI T2 series of the same patient are shown at 11 months (upper image), 15 months (middle image) and 24 months (lower image) after DBS surgery. They indicate an increase of the hyperintense signal changes along the DBS lead over time.

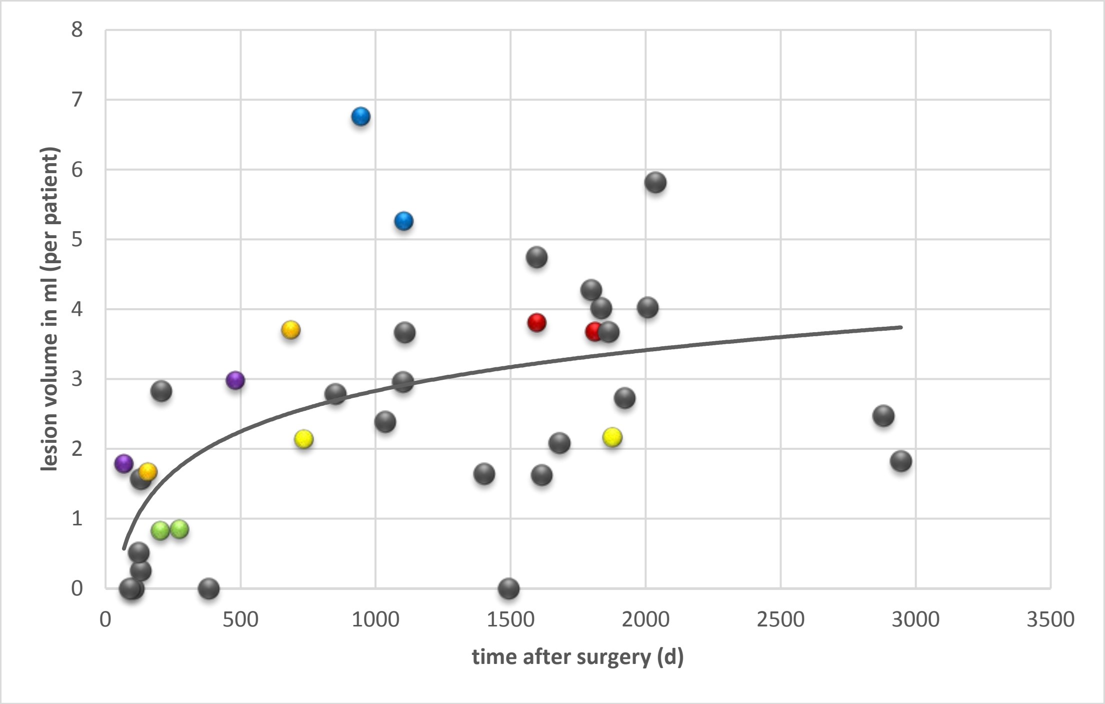

Supplemental Figure 3 | Lesion volume in correlation to time after DBS surgery. 6 patients with more than 1 MRI scan after surgery are highlighted to illustrate the individual time course of lesions in patients with multiple MRI scans in relation to patients with one MRI scan after surgery. Two MRI scans belong to one patient colored green, yellow, dark yellow, blue, red and violet.

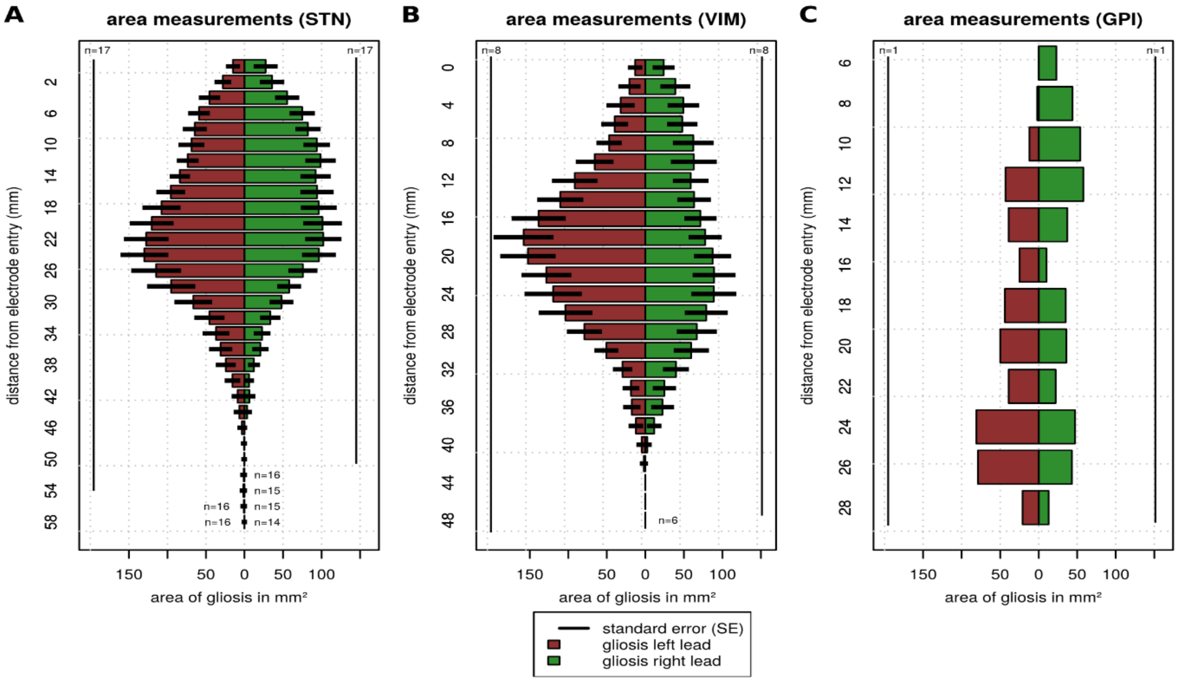

Supplemental Figure 4 | The results of the lesion mapping procedure for three targets (STN, VIM, GPi). The horizontal columns represent the averaged area of the gliosis in mm^2 measured at an orthogonal angle with the DBS trajectory. The results are shown for all left trajectories (red) and right trajectories (green) separately. The number of available measurements (n) is shown at the side of the columns. A shows the results for the STN group. B shows the results for the VIM group and C shows the results of the GPi group. In this group one patient had a gliosis and one patient showed no gliosis.
